# Synergistic Cooperation between Methamphetamine and HIV-1 gsp120 through the P13K/Akt Pathway Induces IL-6 but not IL-8 Expression in Astrocytes

**DOI:** 10.1371/journal.pone.0052060

**Published:** 2012-12-14

**Authors:** Ankit Shah, Peter S. Silverstein, Santosh Kumar, Dhirendra P. Singh, Anil Kumar

**Affiliations:** 1 Division of Pharmacology and Toxicology, School of Pharmacy, University of Missouri-Kansas City, Kansas City, Missouri, United States of America; 2 Department of Ophthalmology and Visual Sciences, University of Nebraska Medical Center, Omaha, Nebraska, United States of America; University of Nebraska Medical Center, United States of America

## Abstract

HIV-1 envelope protein gp120 has been extensively studied for neurotoxic effects that have been attributed to the increased expression of various proinflammatory cytokines in the CNS. Recently we have shown that methamphetamine (MA) also increases expression of proinflammatory cytokines in astrocytes. However, combined effect of gp120 and MA is not known. The present study was undertaken to determine cumulative effect and the mechanism(s)/pathways involved in the functional interaction between gp120 and MA in SVGA astrocytes. Our results clearly suggest that gp120 and MA affect IL-6 but not IL-8 in a synergistic manner and this synergy was mediated by PI3K/Akt and NF-κB pathways. Inhibition of either of these pathways could abrogate the increased expression of IL-6 due to MA or gp120 alone, as well as the increased expression of IL-6 when the astrocytes were treated with both gp120 and MA. These results were confirmed by both, using chemical inhibitors/siRNA as well as western blotting. This study therefore provides novel information regarding the interaction between MA and gp120 in terms of the expression of IL-6 and the mechanisms underlying potential synergy between MA and gp120 in astrocytes.

## Introduction

The incidence of HIV-associated dementia (HAD) has significantly decreased in the post-HAART era. However, HIV-associated neurocognitive disorders (HAND) remains a significant problem [1], [2]. HAND has been generally attributed to the direct as well as indirect effects of viral proteins released from infected microglia and monocytes, the primary targets of HIV in the brain [3,4, reviewed in 5]. In addition, astrocytes can also be infected with the virus [6], [7], [8] resulting into activation of astrocytes and neuronal apoptosis [9].

Of the different HIV-1 proteins, tat and glycoprotein 120 (gp120) have been extensively studied for their neurotoxic potential. Gp120 has been reported to be present in the CNS of HIV-infected patients [10]. Additionally, gp120 is reported to cause increased oxidative stress in the brain [11], increase the BBB permeability [12], [13], increase the cell-death [3], [14], [15] and increase the expression of proinflammatory cytokines and chemokines such as IL-6, IL-8, IL-1β, and CCL5 in astrocytes and neurons [16], [17], [18], [19].

On the other hand a variety of illicit drugs such as cocaine, methamphetamine (MA) and morphine have also been shown to exacerbate HIV-associated neurotoxicity [20], [21], [22], [23]. MA, a potent psycho-stimulant, is known to produce long-lasting dopaminergic insults to the brain [24]. Furthermore, exposure to MA has also been demonstrated to alter BBB integrity [25], [26], induce various cytokines such as TNF-α, IL-1β, IL-8 and IL-6 [27], [28], [29], [30], and oxidative stress [26]. Recently methamphetamine has been shown to act in synergy with gp120 to induce oxidative stress [31]. However it is not known if a synergy exists at the level of induction of proinflammatory cytokines.

The present study was designed to determine whether MA acts with gp120 in a synergistic fashion to induce secretion of proinflammatory cytokines. We examined pathways upstream of NF-κB for involvement in gp120-mediated IL-6 and IL-8 induction as our earlier studies defined the role of NF-κB. We also investigated signaling pathways involved in synergistic production of IL-6.

## Materials and Methods

### Cell culture and reagents

All the experiments were performed with SVGA astrocytes, a clone of SVG astrocytes [32]. SVGA cells were cultured in Dulbecco's Modified Eagle Medium (DMEM) supplemented with 10% Fetal Bovine Serum (FBS), 1% each of Sodium bicarbonate, non-essential amino acids and L-glutamine and 0.1% gentamycin. The cells were cultured in a humidified chamber at 37°C with 5% CO2. The cells were allowed to adhere to the plate before they were used in any of the treatments described below. MA treatments consisted of daily doses of 500 μM MA administered to cells grown in T-75 flasks.

**Figure 1:**
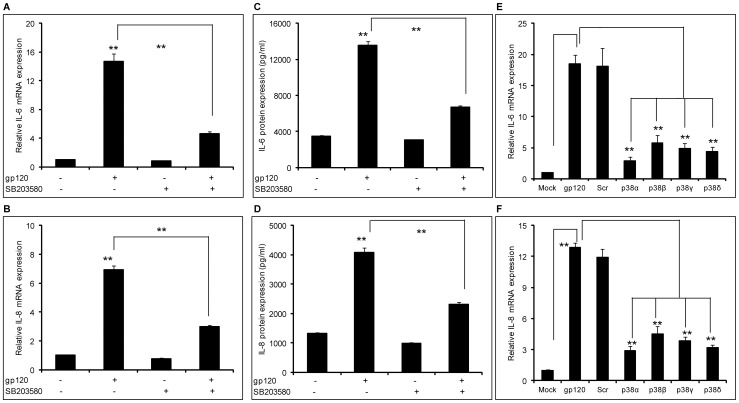
HIV-1 gp120–mediated induction of IL-6 and IL-8 involved the p38 pathway. (A–D) 8×10^5^ SVGA astrocytes, seeded in each well of 6-well plate, were treated with 10 μM of SB203580 1 hour prior to transfection with gp120 plasmid. The cells were transfected in serum-free medium with 2 μg of plasmid expressing gp120. The cells were incubated for 5 hours followed by replacement of the transfection mix with complete medium. The cells were harvested 6 hours post-transfection to determine the mRNA expression levels of IL-6 (A) and IL-8 (B). Identically treated cells were harvested at 24 hours post-transfection to determine protein expression levels of IL-6 (C) and IL-8 (D). SVGA astrocytes were transfected with 50 nM of various siRNA against p38α, p38β, p38γ and p38δ for 48 hours (E–F). Following siRNA transfection, the cells were reseeded at 8×10^5^ cells/well in a 6-well plate and transfected with 2 μg of gp120 plasmid. The cells were harvested 6 hours post-transfection and mRNA expression levels of IL-6 (E) and IL-8 (F) were determined. The bars represent mean ± SE of 3 independent experiments with each treatment in triplicates. The p-value ≤0.01 (**) was considered to be statistically significant using student's t-test.

MA was obtained from Sigma (Sigma-Aldrich, St. Louis, MO). The plasmid encoding gp120 that was used for transfection was originally synthesized by Dr. Park and Dr. Seed [33], [34] and obtained from the NIH AIDS Research and Reference Reagent Program. The inhibitors for the p38 (SB203580), PI3K/Akt (LY294002), JNK (SP600125), ERK1/2 (U0126) and NF-κB (SC514) pathways were obtained from Cayman Chemicals (Cayman Chemicals, Ann Arbor, MI). Antibodies for GFAP (GF5), and IL-6 were obtained from Abcam (Abcam Inc., Cambridge, MA). Vectashield Mounting Medium with DAPI was obtained from Vector laboratories (Vector Laboratories, Burlingame, CA). Pre-designed siRNA for p38α, p38β, p38γ, p38δ and Silencer® Select Negative Control-1 were obtained from Ambion Inc. (Applied Biosystems, Foster City, CA).

**Figure 2:**
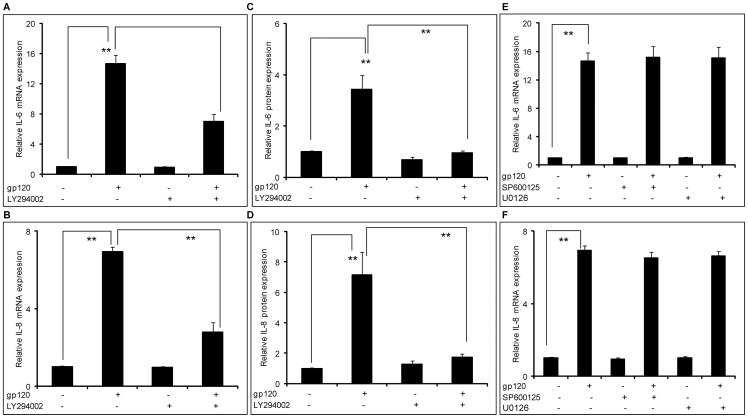
HIV-1 gp120–mediated induction of IL-6 and IL-8 involved the PI3K/Akt pathway. 8×10^5^ SVGA astrocytes, seeded in each well of 6-well plate, were treated with 20 μM of LY294002, a PI3K/Akt inhibitor, 1 hour prior to transfection with gp120. The cells were transfected with 2 μg of gp120 plasmid for 5 hours in serum-free media. Following this, the cells were incubated with complete medium for 6 hours in order to determine the expression levels of IL-6 and IL-8 mRNA (A & B). Identically treated cells were harvested at 24 hours in order to determine the expression levels of IL-6 and IL-8 protein (C & D). The expression levels of IL-6 and IL-8 were compared to the gp120-mediated expression levels of IL-6 and IL-8. Similarly, the cells were treated with SP600125 (a JNK-MAPK inhibitor) or U0126 (an ERK1/2-MAPK inhibitor) 1 hour prior to transfection with gp120 and the expression levels of IL-6 and IL-8 mRNA were determined (E &F). The bars represent mean ± SE of results in 3 different donors with each performed in triplicates. The p-value ≤0.001 (**) was considered statistically significant when calculated using student's t-test.

All the inhibitor treatments were given 1 hour prior to each dose of MA. The cells were harvested 24 hours after the last treatment and total RNA was isolated using a Qiagen RNeasy mini kit. The RNA expression levels of IL-6 and IL-8 were measured using real-time RT-PCR. Treatments with inhibitors involving transfection were performed similarly (i.e. inhibitor added 1 hour prior to the transfection) and the cells were incubated for 5 hours in serum-free transfection media. The transfection media was replaced with media supplemented with 10% serum and incubated for 6 hours in the presence of inhibitor after which the cells were harvested.

**Figure 3:**
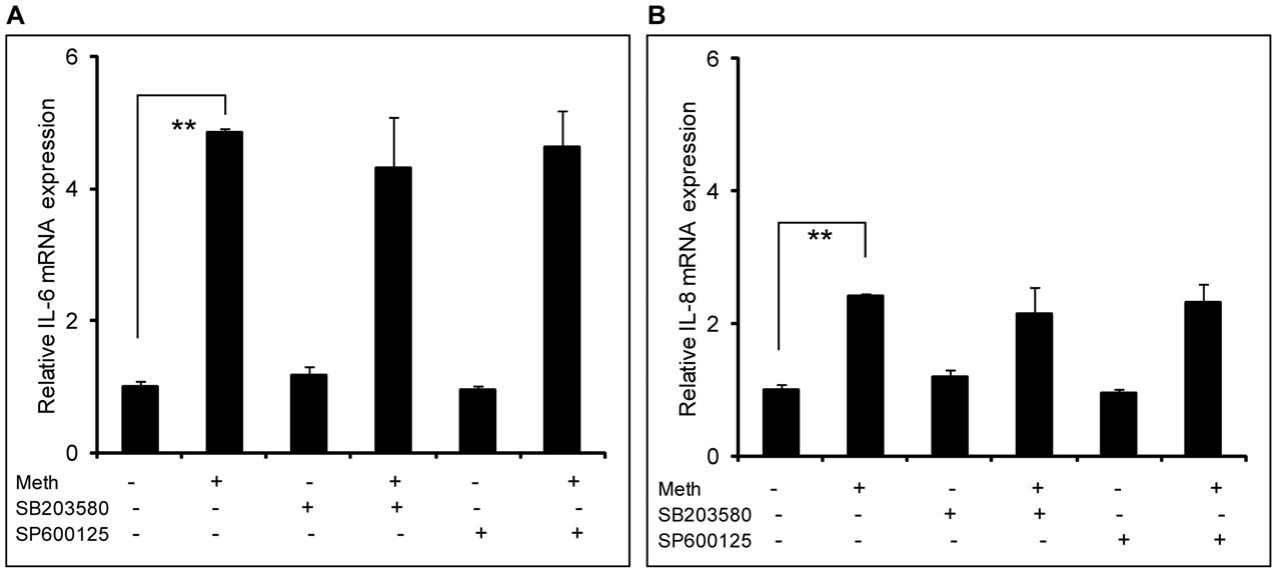
Methamphetamine-mediated induction of IL-6 and IL-8 does not involve the p38-MAPK or JNK-MAPK pathways. SVGA astrocytes were seeded at 8×10^5^ cells in 6-well plate. The cells were treated once every day with 500 μM of MA for 3 days. Cells were treated with inhibitors alone or inhibitors and MA. Treatments with 10 μM of either SB203580 or SP600125 were started 1 hour prior to the MA treatment. The cells were harvested 24 hours after the last treatment of MA and total mRNA was isolated using an RNeasy mini kit. The RNA was analyzed using real time RT-PCR in order to quantify the expression levels of IL-6 (A) and IL-8 (B). Each experiment was repeated at least 3 times with triplicates of each treatment. The p-value ≤0.01 (**) was considered statistically significant when calculated with student's t-test.

### Transfection

SVGA astrocytes were transfected with a plasmid encoding gp120 (pSyngp120-JRFL) using Lipofetamine^TM^ 2000 (Invitrogen Inc. Carlsbad, CA) according to the manufacturer's protocol. Briefly, 8×10^5^ SVGA astrocytes were seeded in 6-well culture plates and grown overnight. On the next day, the cells were washed with PBS and transfected with 2 μg of plasmid encoding gp120 in serum-free media. The cells were incubated with the transfection reagents for 5 hours. After 5 hours, the serum-free media was replaced with complete media containing FBS. The cells were then grown until they were harvested for RNA isolation. For mock-transfected controls, the cells were transfected with an identical vector that did not encode gp120. In the siRNA transfected cells, 50 pmole siRNA against various isoforms of p38 were transfected into the cells 48 hours prior to gp120 transfection. Cells were also transfected with a scrambled sequence of siRNA to control for any non-specific inhibition mediated by siRNA. The cells were incubated with the transfection reagents for 24 hours in serum-free medium after which the transfection reagents were replaced with fresh medium containing FBS and incubated for 24 hours. The cells were trypsinized and reseeded at a density of 8×10^5^ before they were transfected with the plasmid encoding gp120 on the next day. The cells treated with MA and transfected with gp120 were treated with MA every time the reagents were replaced. Similarly, the cells treated with inhibitors were treated with the inhibitors when the medium was replaced.

**Figure 4:**
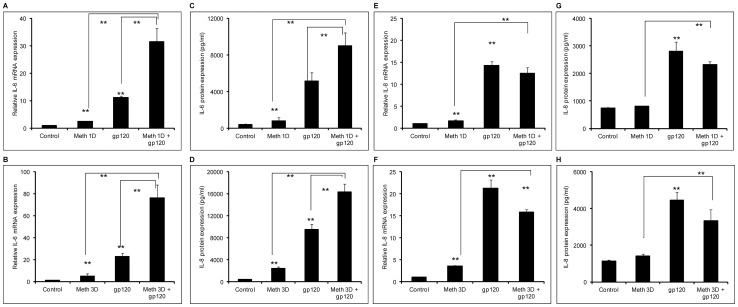
MA exacerbates the induction of IL-6 but not IL-8 by gp120 in astrocytes. SVGA astrocytes were treated with 500 μM of MA for either 24 hours (1 dose) or 72 hours (3 doses). The cells were seeded at 8×10^5^ cells/well in 6-well plates. The cells were transfected with 2 μg of a plasmid expressing gp120. The cells were harvested 6 hours post-transfection for the determination of mRNA levels for IL-6 (A and B) and IL-8 (E and F) and 24 hours post-transfection for protein levels of IL-6 (C and D) and IL-8 (G and H). The response to simultaneous treatment with both MA and gp120 was compared with the levels obtained due to treatment with either MA or gp120 alone. The bars represent the mean ± SE of 3 independent experiments with each treatment in triplicates. The p-values ≤0.01 were considered statistically significant when calculated using student's t-test.

### MA treatment of astrocytes transfected with gp120

Astrocytes were treated with 500 μM of MA once a day for 3 days. The cells were then reseeded in 6-well plates at 8×10^5^ cells/well and transfected with gp120 in either the presence or absence of MA. Astrocytes that were not treated with MA previously were used for appropriate controls. The cells were grown in the transfection reagents for 5 hours followed by incubation in complete media for 6 hours in the presence of MA. Total RNA was isolated to measure the expression of IL-6 and IL-8 at the mRNA level. The cytokines released in the supernatants were measured 48 hours post-transfection using a Bio-Rad multiplex cytokine assay. Cells were also treated with 1 dose of methamphetamine prior to gp120 transfection and cytokine expression at the level of mRNA and protein was measured.

**Figure 5:**
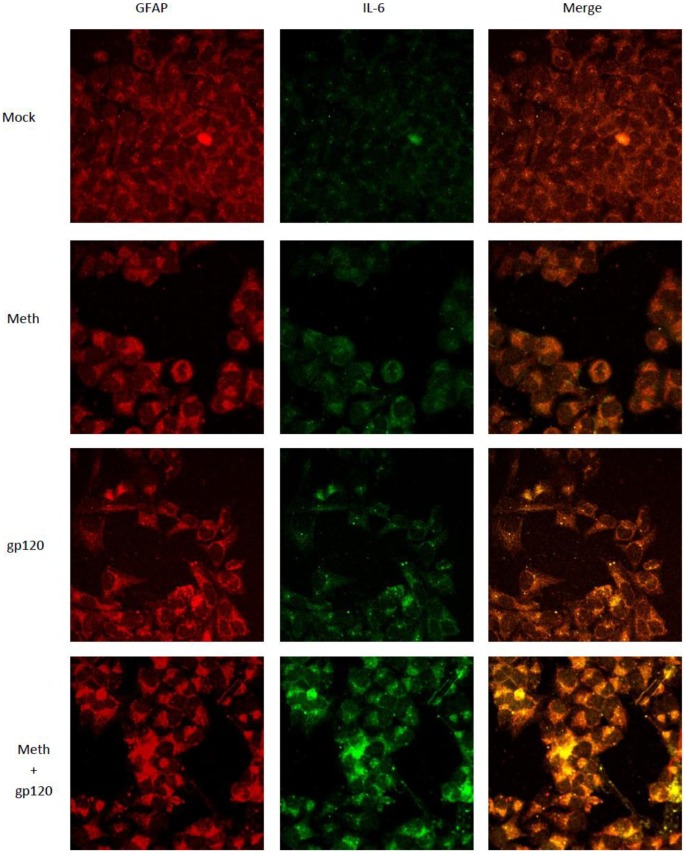
MA and gp120 increase the expression of IL-6 in SVGA astrocytes. SVGA astrocytes were grown on a coverslip and allowed to adhere. The cells were then treated with 500 μM of MA for 24 hours followed by transfection with 2 μg of plasmid encoding gp120. The cytokine release in the supernatant was blocked with the treatment of the cells with GolgiStop^TM^ 6 hours prior to termination. The cells were fixed 24 hours post-transfection with 1∶1 methanol:acetone. The cells were permeabilized with 0.1% PBS-Triton X-100 for 10 min and blocked with 1% BSA in PBS. After blocking, the coverslip was incubated with a primary rabbit antibody against IL-6 and a primary mouse antibody against GFAP for 1 hour. This was followed by washes with PBST and incubation with fluorescently labeled secondary antibodies for 1 hour. Anti-rabbit Alexafluor488 [15] and anti-mouse Alexafluor555 (red) were used to detect IL-6 and GFAP, respectively. The cells were washed with PBST and the coverslips were mounted on a glass slide using mounting solution. The cells were observed using confocal microscopy. The images shown here, obtained using EZ C1 confocal microscope software, are 60X magnification.

### Real time RT-PCR

The mRNA expression levels of IL-6 and IL-8 were measured by amplifying the total RNA using real time RT-PCR. Briefly, total RNA was isolated using an RNeasy mini kit (QIAGEN, Valencia, CA) as per the manufacturer's protocol. The RNA was then reverse transcribed using reverse transcriptase (RT) at 50°C for 30 min. The cDNA obtained was denatured at 95°C for 15 min and amplified for 45 cycles (95°C for 15 sec, 57.5°C for 1 min) using primers (Forward: 5′-GGT ACA TCC TCG ACG GCA TC-3′ and Reverse: 5′-CCA GTG CCT CTT TGC TGC TT-3′) and probe (5′-FAM-CAG CCC TGA GAA AGG AGA CAT GTA ACA GGA GGT AA-3′-BHQ1) for IL-6 and another set of primers (Forward: 5′-CTC TTG GCA GCC TTC CTG ATT-3′ and Reverse: 5′-TAT GCA CTG ACA TCT AAG TTC TTT AGC A- 3′) and probe (5′-FAM-CTT GGC AAA ACT GCA CCT TCA CAC AGA-3′BHQ1) for IL-8. Hypoxanthine-guanine phosphoribosyl transferase (HPRT) mRNA was amplified and IL-6 expression was normalized to this housekeeping gene. The conditions and primers used to determine HPRT levels were as follows: forward primer: 5′-GCT TTC CTT GGT CAG GCA GTA-3′; reverse primer: 5′-CCA ACA CTT CGT GGR GTC CTT T-3′; reverse transcription at 50°C for 30 m, 95°C for 15 m and 45 cycles at 95°C for 15 sec and 55°C for 30°sec. HPRT amplification products were detected using SYBR green. The data was analyzed using the 2^−ΔΔCt^ method as described previously [35].

**Figure 6:**
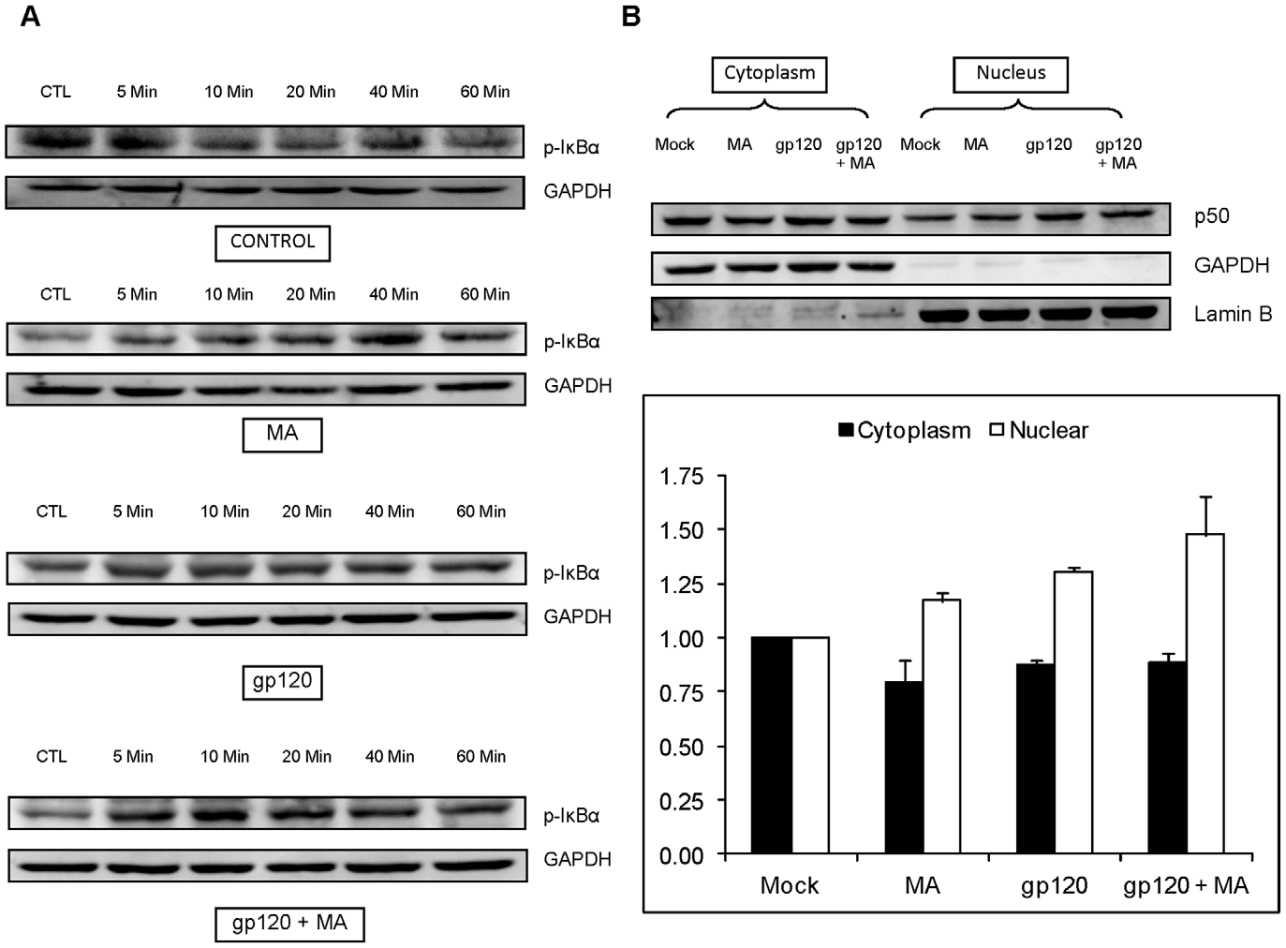
gp120 and/or MA activates IκBα and increases the translocation of p50 in SVGA astrocytes. (A) SVGA astrocytes were treated with 500 μM MA and/or 50 ng/ml recombinant gp120 protein for 0, 5, 10, 20, 40 and 60 minutes and the whole cell lysate was prepared by lysing the cells with RIPA buffer. The proteins were separated using SDS-PAGE electrophoresis as mentioned in the Materials and Methods section. The p-IκBα was determined in western blotting. GAPDH was used as housekeeping control. (B) SVGA astrocytes were treated with MA and/or transfected with gp120 for 6 hours. The translocation of p50 was measured from cytoplasm to nucleus as described in the Materials and methods. GAPDH was again used as loading control for cytoplasmic extracts and LaminB was used as loading control for nuclear extracts. The bar charts below the representative images show the densitometry values corresponding to the respective treatments. The housekeeping proteins were used to normalize the protein expressions of various genes.

**Figure 7:**
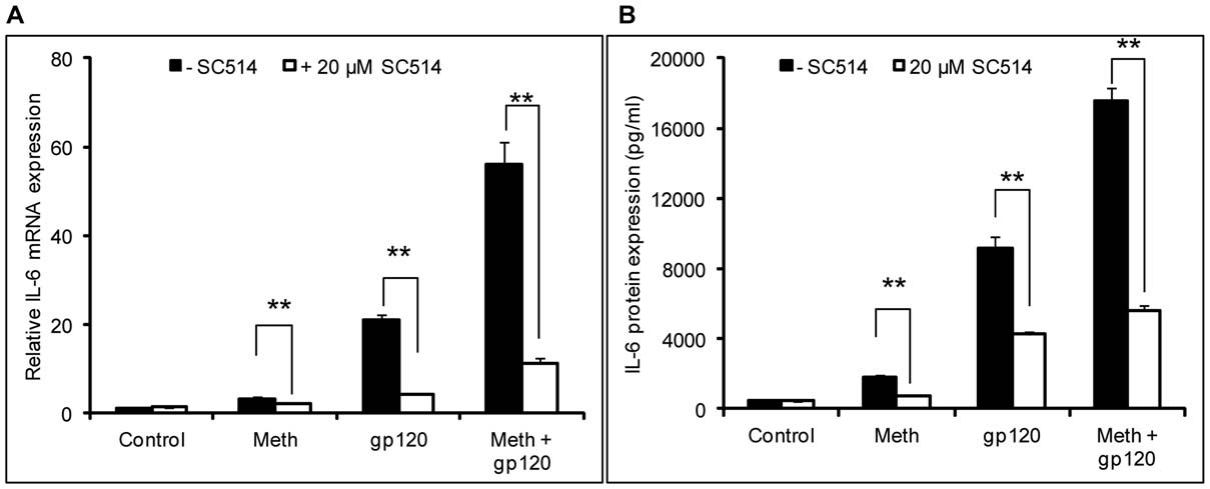
The NF-κB pathway is involved in the induction of IL-6 by MA and gp120. SVGA astrocytes were treated with 500 μM of MA once a day for 3 days for MA treatment. The cells were then seeded in 6-well plates with 8×10^5^ cells and transfected with 2 μg of plasmid expressing gp120. Untreated cells were also transfected with 2 μg of plasmid expressing gp120. For inhibitor treatments, cells were treated with the specific inhibitors for the NF-κB pathway (SC514) 1 hour prior to addition of MA and/or gp120. The cells were harvested 6 hours post-transfection to quantify the mRNA expression levels of IL-6 in the presence of SC514 (A). In order to determine the levels of IL-6 protein, the cell culture supernatants were collected 24 hours post-transfection (B). Each bar shows the mean ± SE of 3 independent experiment with each performed in triplicates. The p-value of ≤0.01 (**) was considered to be statistically significant as calculated using student's t-test.

### Multiplex protein analysis

Cell culture supernatants were collected at 48 hours post-transfection and stored at −80°C until analyzed. Cytokine expression in supernatants was measured using the Bio-Plex system (Life Science Research, Hercules, CA) and its associated Bio-Plex Manager 5.0 software. Protein levels in the supernatant were determined using a Bio-Rad multiplex kit and analyzed using the 5-PL standard curve method. Briefly, premixed BioPlex bead stock was added to the pre-wetted filter plate. Standards or cell culture supernatants were then added, incubated while shaking continuously for 30 min, and the excess was washed off. Detection antibodies were added and again incubated for 30 min and then excess was removed with wash buffer. Finally, phycoerythrin (PE)-conjugated streptavidin was added and the beads were then suspended in assay buffer and analyzed for cytokine expression.

**Figure 8:**
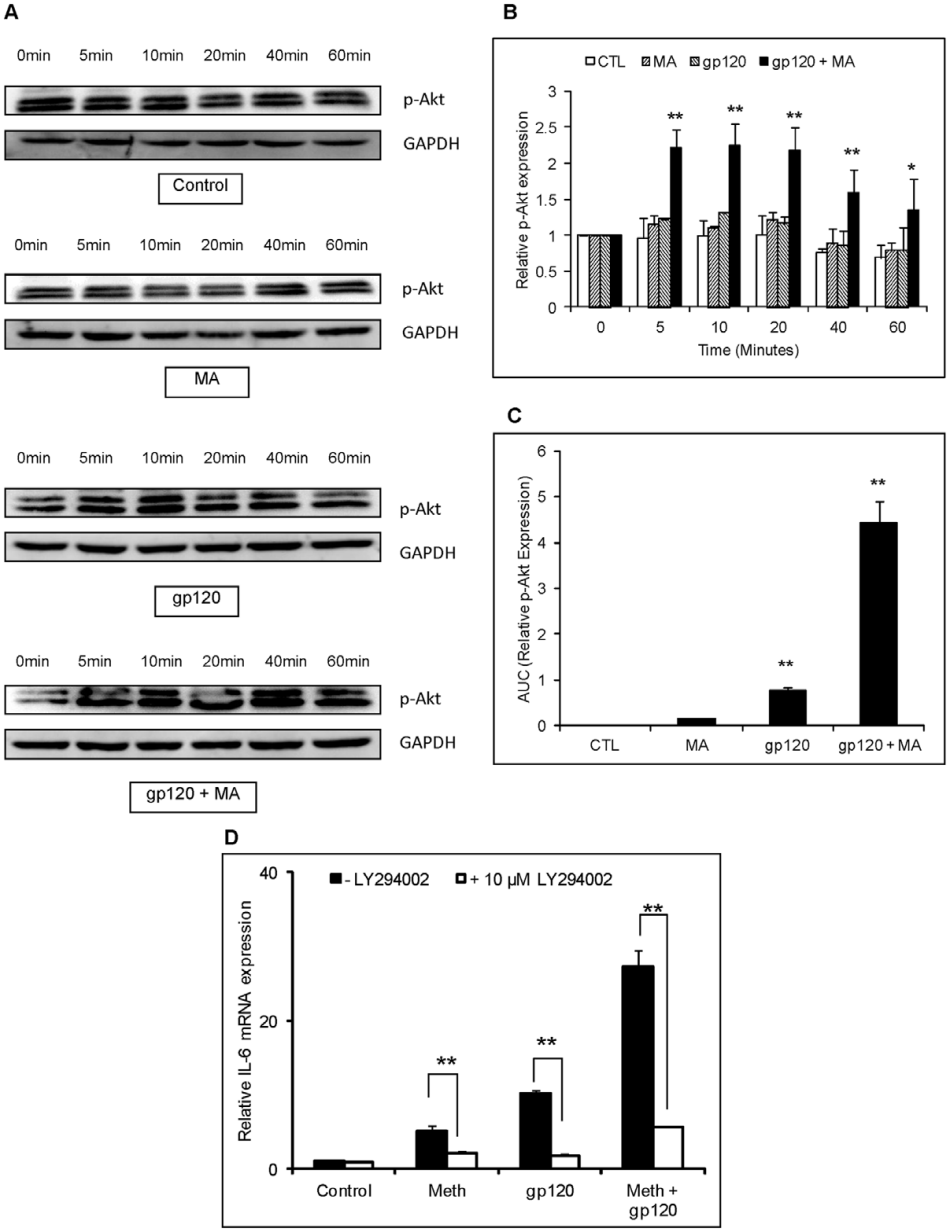
gp120 and/or MA activate PI3K/Akt signaling in astrocytes. SVGA astrocytes were treated with 500 μM MA and/or 50 ng/ml recombinant gp120 protein for 0, 5, 10, 20, 40 and 60 minutes. The cells were lysed using RIPA buffer and homogenized to prepare whole-cell lysate. The protein was electrophoresed and p-Akt was detected in western blotting. GAPDH was used as a loading control (A). The bar chart represents the normalized densitometry values measured using GAPDH as control. The expression of p-Akt observed at 0 min was considered as basal expression (B). The area under the curve of the normalized p-Akt expression was calculated by adding the expression levels at different time-points shown in (A) and (B) in the kinetics. The values were normalized with untreated control as basal expression levels (C). For inhibitor treatments, cells were treated with the specific inhibitors for the PI3K/Akt pathway (LY294002) 1 hour prior to addition of MA and/or gp120. The cells were harvested 6 hours post-transfection to quantify the mRNA expression levels of IL-6 (D).

### Immunocytochemistry

In order to observe the intracellular expression of IL-6, SVGA cells were immunostained and observed under a confocal microscope. Briefly, 3.5×10^5^ SVGA cells were seeded in a 12 well plate containing glass coverslips at the bottom of each well. The cells were treated as indicated and incubated for 24 hours. GolgiStop^TM^ (BD biosciences, San Jose) at a concentration of 1 mg/ml was added 6 hours prior to the termination of the indicated treatment in order to prevent the release of cytokine from the cells. Upon termination, the cells were fixed with 1∶1 mixture of ice-cold acetone: methanol for 20 min and incubated at −20°C. Following fixation, the cells were washed 3X with PBS and permeabilized with PBS containing 0.1% Triton X100 (PBST) for 20 min. The coverslips were then blocked with 1% BSA in PBST for 30 min. This was followed by incubation with a cocktail of rabbit anti-IL-6 (1∶500) and mouse anti-GFAP (GF5) (1∶1000) primary antibodies for 1 hour in a humidified chamber at room temperature followed by 3 washes with PBS for 5 min. The coverslips were then incubated with fluorescent-labeled secondary antibodies against mouse IgG (conjugated with Alexa Fluor 555) (1∶1000) and rabbit IgG (conjugated with Alexa Fluor 488) (1∶1000) for 1 hour in a dark humidified chamber at room temperature. The coverslips were then washed 3X with PBS for 5 min each. Finally, the coverslips were mounted on glass slides using 10 μl vectashield containing DAPI. The slides were analyzed using confocal microscopy in order to obtain fluorescent images using a Nikon Eclipse E800 confocal microscope (Nikon Instruments, Melville, NY). The images were captured using a 60X zoom lens.

**Figure 9:**
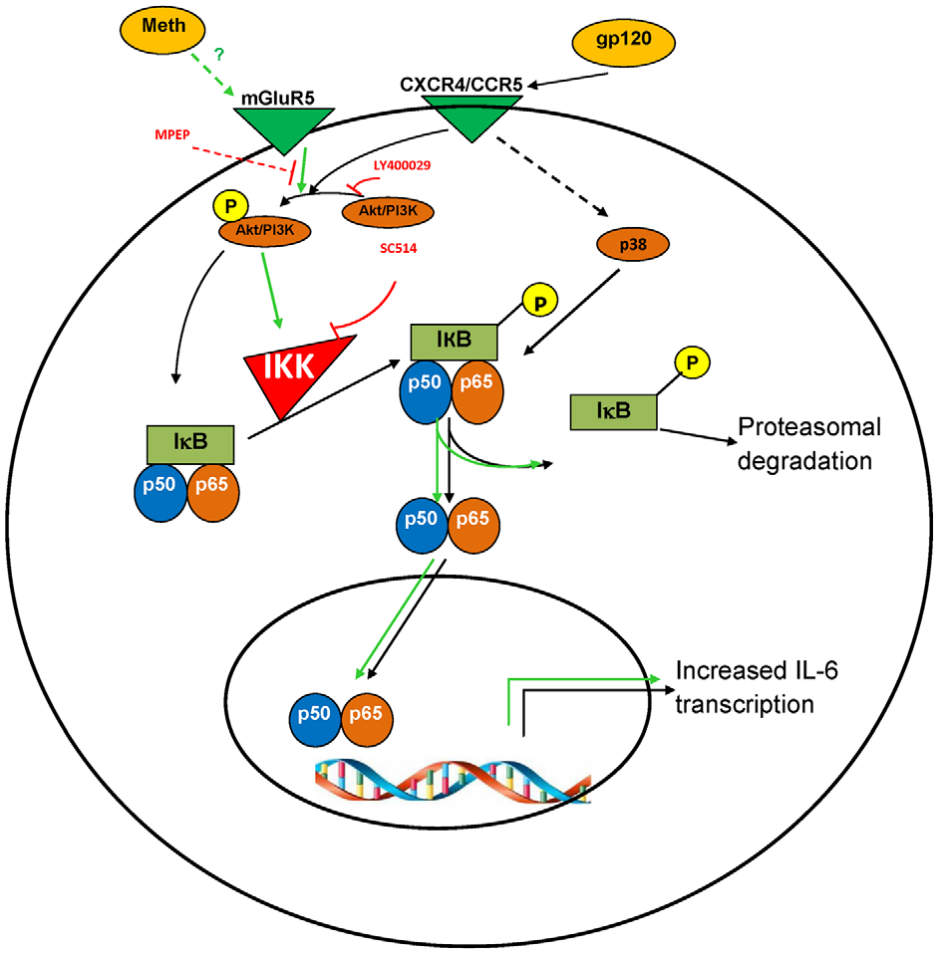
Schematic illustrations of the signaling pathways that mediate the induction of IL-6 by MA and gp120 in astrocytes. Treatment of astrocytes with gp120 results in the binding of gp120 by the CCR5 or CXCR4 chemokine receptors and the subsequent activation of PI3K/Akt and p38-MAPK. The activation of these two pathways may lead to increased activation of NF-κB. The activated NF-κB then translocates into the nucleus and increases the transcription of IL-6. Treatment of astrocytes with MA results in activation of metabotropic glutamate receptor 5 (mGluR5). The mechanism of mGluR5 activation by MA is unclear, but one possibility is that the increase in extracellular glutamate caused by MA treatment may activate the receptor. mGluR5 then activates the PI3K/Akt pathway [30] which can lead to activation of NF-κB and increased expression of IL-6. Thus, gp120 and MA can induce higher expression levels of IL-6 as compared to the levels observed in the presence of either agent alone.

### Western Blotting

The SVGA cells were treated with either MA or gp120 alone or both of them together for various lengths of time and lysed using (Radio Immunoprecipitation Assay) RIPA buffer. The whole-cell lysates were homogenized and spun for 10 min at 10000xg to remove debris. The nuclear and cytoplasmic proteins were separated using cytoplasmic buffer (10 mM HEPES, 50 mM NaCl, 0.5 M sucrose, 0.1 mM EDTA and 0.5% TritonX100) and nuclear buffer (10 mM HEPES, 500 mM NaCl, 0.1 mM EDTA, 0.1 mM EGTA and 0.1% IGPAL) as described previously [18]. The protein concentration was measured using Bradford's assay and 30 μg protein was loaded in each well for electrophoresis. The proteins were separated at 80V for 3 hours on 10% polyacrylamide gel and transferred on to PVDF membrane at 350 mAmp for 90 minutes. The membranes were probed with various primary and secondary antibodies and the bands were detected using BM Chemiluminescence Western Blotting Substrate (POD) (Roche Applied Sciences, Indianapolis, IN). The bands were quantified using FlourChem HD2 software (Alpha Innotech, San Leandro, CA) and the intensities were normalized with GAPDH for whole-cell lysate or cytoplasmic protein; and, with laminB for nuclear proteins.

### Statistical Analysis

Statistical analysis was performed using the Student's t-test assuming equal variance. Results were based on 3 separate experiments with each experiment conducted in triplicates. Results were considered statistically significant if p≤0.05. All the data presented in the bar charts are presented as mean ± SE of 3 independent experiments.

## Results

A recent report suggesting the role of gp120 in overexpression of pro-inflammatory cytokines such as IL-6 in astrocyte cell line as well as human fetal astrocytes [36] clearly underlines the need to study the mechanisms involved in gp120-mediated inflammatory responses in astrocytes. In our earlier studies, we have shown that both MA and gp120, when administered alone, can increase the expression of proinflammatory cytokines/chemokines such as IL-6, CCL5 and IL-8 in astrocytes [17], [18], [30]. We have also shown involvement of NF-κB in both gp120 and MA mediated induction and PI3K/Akt pathways were found to be involved in only MA-mediated induction of IL-6 and IL-8 [18], [30]. In the present study we identified other pathways involved in mediating the effect of gp120 and MA in IL-6 and IL-8 induction in astrocytes.

### HIV-1 gp120 – mediated expression of IL-6 and IL-8 involves the p38 and PI3K/Akt pathways

In our previous studies, we demonstrated that gp120-mediated induction of IL-6, IL-8 and CCL5 is NF-κB-dependent [17], [18]. In order to further investigate the mechanisms involved in the induction of these cytokines, we investigated the possible roles of the p38-MAPK, PI3K/Akt, ERK1/2- MAPK, and JNK-MAPK pathways. SVGA astrocytes were treated with 10 μM of p38-MAPK inhibitor (SB203580) 1 hour prior to gp120 transfection. The dose of SB203580 was determined based on the cell viability as determined by trypan blue staining (data not shown). Treatment of the cells with SB203580 could reduce gp120-mediated expression of both IL-6 (Fig. 1A) and IL-8 (Fig. 1B) by 74.4±2.2% and 65.6±1.9%, respectively. We also determined the protein levels of the cytokines released in the supernatants using a multiplex cytokine bead assay and found similar results that SB203580 was able to reduce gp120-mediated expression of IL-6 (Fig. 1C) by 50.6±1.6% and IL-8 (Fig. 1D) by 43.2±1.5%. In order to further confirm the involvement of p38 in gp120-mediated induction of IL-6 and IL-8, we used siRNA for various isoforms of p38. All of the p38-specific siRNAs could inhibit gp120-mediated mRNA expression of IL-6 and IL-8 by more than 70% as shown in Fig. 1E and 1F, respectively. Together, these results suggest involvement of the p38-MAPK pathway in gp120-mediated induction of IL-6 and IL-8. In addition to p38α, which leads to activation of the transcription factor NF-κB, we found that p38β, p38γ and p38δ are also involved in gp120-mediated induction of IL-6. The other isoforms of p38 may activate additional transcription factors such as STAT1, CHOP, Max and ATF2 [reviewed in 37].

Having established the involvement of p38-MAPK pathway, we wished to examine signaling events upstream of the NF-κB pathway. Involvement of the PI3K/Akt pathway in inducing IL-6 in an NF-κB-mediated pathway has been reported in microglia [38]. Thus, we wished to study the possible role of the PI3K/Akt pathway in gp120-mediated induction of IL-6 and IL-8. In order to do so, we used a specific inhibitor LY294002 to block the PI3K/Akt pathway. SVGA astrocytes were treated with 20 μM of LY294002 1 hour prior to gp120 transfection. The dose of LY294002 was determined based on the cell viability as determined by trypan blue staining (data not shown). The abrogation of gp120-mediated induction of IL-6 and IL-8 at the mRNA level was measured using real time RT-PCR. The inhibitor could reduce gp120-mediated expression of IL-6 by 56.3±7.5% (Fig. 2A) and IL-8 by 70.1±8.5% (Fig. 2B). Thus, our results suggest involvement of both p38-MAPK and PI3K/Akt pathways in gp120-mediated increased expression of IL-6 and IL-8 in astrocytes. Furthermore, these results were confirmed at the protein level as shown in Fig. 2C and 2D for IL-6 and IL-8, respectively. The PI3K/Akt inhibitor abrogated gp120-mediated IL-6 and IL-8 expression at the level of protein by more than 90%. We also investigated the ERK1/2-MAPK and JNK-MAPK pathways to study their roles in the gp120-mediated induction of IL-6 and IL-8. As in the previous experiments, we used 20 μM of ERK1/2-MAPK inhibitor, U0126 and JNK-MAPK inhibitor, SP600125. As shown in Fig. 2E and 2F, both the inhibitors failed to abrogate the gp120-mediated expression of IL-6 and IL-8.

### MA-mediated induction of IL-6 and IL-8 does not involve the p38-MAPK or JNK-MAPK pathways

In our prior study, we showed that the MA-mediated increase in the expression of IL-6 and IL-8 involved the NF-κB and the PI3K/Akt pathways [30]. However, in making a determination as to the mechanisms responsible for MA-gp120 interactions, it is important to explore other pathways that may be involved in the interaction. In order to further explore the signaling mechanism(s) responsible for MA-mediated expression of proinflammatory cytokines, we investigated the potential roles of the JNK-MAPK and p38-MAPK pathways, as both pathways have the capability of activating the NF-κB transcription factor. We treated SVGA astrocytes with 500 μM MA once a day for 3 days. The dose of methamphetamine was calculated using blood levels of methamphetamine in conjunction with tissue/serum compartmentalization ratios reported in the literature [39], [40], [41]. It has been previously extrapolated that 250 mg-1g binge methamphetamine administration as single dose could produce between 164–776 µM concentrations in the brain [41]. The cells were treated with 10 μM of specific inhibitors for either JNK-MAPK (SP600125) or p38-MAPK (SB203580) pathway as described in materials and methods. As seen in Figure-3, neither of the inhibitors could abrogate the MA-mediated expression of IL-6 (Fig. 3A) and IL-8 mRNA (Fig. 3B). Thus, we conclude that neither the JNK-MAPK nor the p38-MAPK pathways are involved in the MA-mediated induction of cytokines/chemokines.

### MA exacerbates gp120-mediated induction of IL-6 but not IL-8 in astrocytes

Taken together, the results shown in the present study along with our prior studies with either MA or gp120 administered alone, suggest that there are overlapping mechanisms involved in the induction of cytokine levels in astrocytes. Furthermore, MA has been shown to exacerbate the toxicity of tat and gp120 via multiple mechanisms such as increasing oxidative stress [31], dopaminergic insult [42], [43], and alteration in BBB integrity [25]. Therefore, we hypothesized that there might be interaction between pathways governing induction of cytokines by MA and gp120 in astrocytes. Our data demonstrate that astrocytes treated with both MA and gp120 increased the expressions of IL-6 to levels that are much higher than when cells were treated with either agent alone. This was observed for astrocytes treated with either 1 dose or 3 doses of MA given over 3 days at the levels of both, mRNA (Fig. 4A and 4B) and protein (Fig. 4C and 4D). However, we did not observe the same effects upon IL-8 expression either at the levels of mRNA (Fig. 4E and 4F) or protein (Fig. 4G and 4H). Although, astrocytes treated with either MA or gp120 alone increased the level of IL-8 expression, treatment with both MA and gp120 simultaneously did not show levels of expression that would suggest synergy. Furthermore, astrocytes treated with MA and gp120 for 3 days exhibited lower expression levels of IL-8 mRNA (p<0.05) as compared to astrocytes treated with gp120 alone. However, when IL-8 expression in these two groups was compared at the level of protein, the difference between the groups was not significantly different. We therefore did not pursue the mechanism(s) involved in regulating IL-8 expression in response to simultaneous treatment with both MA and gp120.

In order to confirm these results, we also used immunofluorescent staining as described in the materials and methods. As shown in Figure 5 treatment of cells with either MA or gp120 increased the expression of IL-6. However, when cells were treated with both gp120 and MA the levels of IL-6 were much higher than the levels observed when treatment was with either agent alone. Thus, our results suggest a possible synergistic interaction between MA and gp120 in terms of the expression of IL-6 at the levels of both mRNA as well as protein.

### The PI3K/Akt and NF-κB pathways are involved in the induction of IL-6 expression by MA and gp120

Having established that MA exacerbates the expression of IL-6 induced by gp120, we wished to investigate the mechanisms governing this interaction. We hypothesized that the NF-κB pathway was likely to be involved in this interaction because both MA and gp120 utilized this transcription factor to increase expression of IL-6. In order to confirm the signaling mechanism, we measured the p-IκBα in the whole-cell lysates and translocation of p50 from the cytoplasmic to nuclear compartment. In order to measure the phosphorylation of IκBα, SVGA cells were treated either with MA or recombinant gp120 alone or gp120+MA for 0, 5, 10, 20, 40 and 60 min. The whole-cell lysates were prepared and the expression of p-IκB-α was measured using western blotting (Fig. 6A). The phosphorylation of IκBα was observed within 5 minutes with peak at 40 min for MA and 10 min for gp120. Cells treated with gp120 and MA showed increase in p-IκBα at 5 min and the expressions remained significantly high throughout observation period of 60 min. We have already shown in our previous study that both MA and gp120 can independently increase the translocation of p50 within 3 hours and 6 hours, respectively [18], [30]. In order to measure the translocation of p50, SVGA astrocytes were treated with either MA or gp120-transfection alone or both MA and gp120-transfection for 6 hours and the p50 expression was measured using western-blotting (Fig. 6B). The translocation of p50 was observed in the samples treated with MA or gp120 and MA+gp120, however, there was no significant difference between gp120 transfection and gp120 transfection/MA treatment. Furthermore, SVGA astrocytes were treated with 20 μM SC514 1 hour prior to the MA treatments and gp120 transfection. The cells were harvested 6 hours post-transfection as described in materials and methods. Treatment with an NF-κB antagonist abrogated MA and gp120 –mediated expression of IL-6 when cells were treated with either agent alone. Furthermore, it also abrogated the increase of IL-6 induced by simultaneous treatment with both MA and gp120 at the levels of mRNA (Fig. 7A) and protein (Fig. 7B).

We also investigated the potential role of PI3K/Akt in the increase of IL-6 expression since both MA and gp120 utilized PI3K/Akt in inducing the expression of IL-6. SVGA astrocytes were treated with either MA or gp120 alone or both gp120 and MA, and the whole-cell lysates were collected after 0, 5, 10, 20, 40 and 60 min. The proteins were separated using SDS-PAGE electrophoresis and the expression of phosphorylated Akt was measured in western blot assay. Both gp120 and MA showed increased expression of p-Akt with MA and gp120 showing higher expressions of p-Akt than either of the agents alone (Fig. 8A). Furthermore, the levels of PI3K/Akt-activation was observed to be more than additive in the astrocytes, treated with gp120 and MA as opposed to those treated with either MA or gp120 alone (Fig. 8B). Additionally, the area under curve for gp120 and MA was significantly higher than either MA or gp120 alone (Fig. 8C). Furthermore, LY294002 (an inhibitor of the PI3K/Akt pathway) abrogated IL-6 expression (Fig. 8D). Taken together, these results suggest that both the NF-κB and PI3K/Akt pathways are responsible for the increase of IL-6 levels observed with the combined treatment with gp120 and MA in astrocytes.

## Discussion

One of the features of many CNS diseases is the presence of elevated levels of cytokines. For example, increased levels of cytokines and chemokines have been found in CNS diseases such as Parkinson's disease (PD), Alzheimer's disease (AD) and Multiple Sclerosis (MS) [reviewed in 44,45]. Altered levels of cytokines and chemokines also play an important role in the pathology of HAND [reviewed in 46]. MA, and most other drugs of abuse, are associated with both a greater risk in the acquisition of HIV infection and more rapid disease progression. Although, several reports have documented the deleterious effects of various drugs of abuse in the CNS, the mechanism by which these drugs exacerbate HIV infection of the CNS remains unclear. In an early study, Nath et al. reported that an HIV-infected abuser of MA and cocaine exhibited CNS complications that developed more rapidly and was more severe than those observed in non-abusing HIV+ patients [47]. MA has also been found to act synergistically with Tat in producing CNS damage in a rat model [reviewed in 48]. Furthermore, studies have documented the effects of MA on increased viral load/infectivity in different biological compartments using either *in vitro* or *in vivo* models [49], [50], [51], [52].

We hypothesized that, MA and gp120 might exacerbate the neuroinflammatory response via overlapping signaling pathways. Our previous work demonstrated that HIV gp120 induces IL-6 expression in astrocytes through an NF-κB-dependent mechanism. In the present work we have shown that the PI3K/Akt and p38 MAPK pathways also play a role in gp120-mediated induction of IL-6. The involvement of the p38MAPK pathway was confirmed through the use of siRNA targeted against p38 homologs. This is in accordance with the earlier reports that documented the role of the p38MAPK pathway in gp120-induced neurotoxicity and release of neurotoxins in monocytes and mixed cerebrocortical cultures [3], [53]. Using a rat astrocyte model, Yang et al. has shown that gp120-mediated cellular toxicity involves the MAPK pathway [22]. However, unlike the report from Yang et al., in our system we did not detect any involvement of either the ERK1/2-MAPK or JNK-MAPK pathways in the induction of IL-6 by gp120. This discrimination could be attributed to the fact that source of astrocytes in our study was human and not rat as used in previous study. It is also possible that the ERK1/2-MAPK and JNK-MAPK pathways may have a role in gp120-mediated apoptosis of astrocytes, but not in the increased expression of IL-6 and IL-8. In the present study, we also demonstrate a role for the PI3K/Akt pathway in gp120-mediated induction of IL-6 and IL-8. The PI3K/Akt pathway, acting in conjunction with other factors, is known to increase the activation of the NF-κB pathway by phosphorylation of IKK [54], [55]. Similarly, activation of PI3K/Akt can also lead to the activation of p38-MAPK pathway [56]. Thus, our study indicates involvement of both the PI3K/Akt and the p38-MAPK pathways in the induction of cytokines by gp120. Both p38-MAPK and PI3K/Akt may function to further activate the NF-κB pathway; as such an activity has been described for both of these pathways [55], [57]. Furthermore, numerous studies have shown that NF-κB can promote transcription of various cytokines and chemokines. Together, our study provides a detailed insight into the mechanisms in gp120-mediated induction of cytokines.

We have shown that MA can increase the expression of IL-6 and IL-8 in astrocytes [30]. We therefore, wished to investigate the combined effects of gp120 and MA on the induction of proinflammatory cytokines in astrocytes. The present study shows for the first time that, when administered simultaneously, gp120 and MA can increase the expression of IL-6 to levels that are significantly higher than that induced by either agent alone. Thus, our data suggests that gp120 and MA act synergistically to induce IL-6. Our earlier reports, along with this study, show that the effects of both gp120 and MA are mediated by the PI3K/Akt and the NF-κB pathways. Therefore, we hypothesized that these two pathways interact in tandem to increase expression of IL-6 after simultaneous administration of MA and gp120 in astrocytes. Indeed, we found increased phosphorylation of Akt and IκB-α upon treatments with either MA or gp120 alone as well as both of them together. The phosphorylation of Akt was observed to be higher in the cells treated with MA and gp120 together. Furthermore, the p-IκB-α levels were observed to be not only higher but also the activation was observed earlier than the cells treated with either MA or gp120 alone. The involvement of either PI3K/Akt or NF-κB was further confirmed by the fact that specific inhibitors abrogated the synergy in MA and gp120-mediated IL-6 production. Although gp120 was found to increase the expression of IL-6 through activation of the p38-MAPK pathway, the MA-mediated increase of IL-6 did not involve the p38-MAPK pathway. Our work confirms a previous study using a human neuroblastoma cell line that showed that MA exposure did not activate the p38-MAPK pathway in these cells [58]. Therefore, we ruled out the possibility of the involvement of the p38-MAPK pathway in the MA-mediated exacerbation of the expression levels of IL-6 induced by gp120. On the contrary we did not observe synergy in IL-8 production whereas both MA and gp120 individually increased the expression of IL-8 in astrocytes.

In conclusion, we have demonstrated that both MA and gp120 could induce the expression of IL-6 in astrocytes by utilizing common signaling pathways. The results from this study suggest a model for synergistic IL-6 production in astrocytes (Figure 9). When in the CNS, MA and gp120 can activate the PI3K/Akt pathway via their action on mGluR5 [59], [60], [61] and the HIV-1 co-receptors that bind gp120 (i.e. CXCR4 or CCR5). The activation of PI3K/Akt can further activate the NF-κB pathway via phosphorylation of IKK, which can lead to the increased expression of IL-6. Additionally, the p38-MAPK pathway is involved in the increased expression levels of IL-6 due to gp120. The p38-MAPK pathway is known to activate the NF-κB pathway in addition to other transcription factors such as AP-1, STAT1 and ATF2. However, this pathway may not be involved in the MA-gp120 interaction that results in increased expression of IL-6 in astrocytes. This study, therefore, provides key information with regard to molecular pathways involved in MA-mediated exacerbation of the induction of IL-6 by gp120. Previously, MA has been shown to potentiate the oxidative stress induced by HIV-1 proteins, including gp120, which may result in disruption of tight junction proteins in the BBB [25], [26]. Additionally, MA has been shown to act as a co-factor in the alteration of BBB permeability induced by gp120 [31]. Taken together with the results of the present work, these findings indicate possible synergistic interactions between MA and gp120 at several different levels that may exacerbate the neuro-toxicity related to neuroAIDS. Understanding the mechanisms involved in these interactions will be of help in development of therapeutic strategies for the treatment of HAND among MA abusers.
