# Correction: Synergistic Cooperation between Methamphetamine and HIV-1 gsp120 through the P13K/Akt Pathway Induces IL-6 but not IL-8 Expression in Astrocytes

**DOI:** 10.1371/annotation/23d0ff01-4f27-4ecd-9a8e-9018d2ad0ae0

**Published:** 2013-10-01

**Authors:** Ankit Shah, Peter S. Silverstein, Santosh Kumar, Dhirendra P. Singh, Anil Kumar

There is an error in the title. "gp120" was incorrectly labeled as "gsp120."

The correct title is: Synergistic Cooperation between Methamphetamine and HIV-1 gp120 through the P13K/Akt Pathway Induces IL-6 but not IL-8 Expression in Astrocytes

The correct citation is: Shah A, Silverstein PS, Kumar S, Singh DP, Kumar A (2012) Synergistic Cooperation between Methamphetamine and HIV-1 gp120 through the P13K/Akt Pathway Induces IL-6 but not IL-8 Expression in Astrocytes. PLoS ONE 7(12): e52060. doi:10.1371/journal.pone.0052060 

